# Imported *Haycocknema perplexum* Infection, United States^1^

**DOI:** 10.3201/eid2811.220286

**Published:** 2022-11

**Authors:** Bobbi S. Pritt, Blaine A. Mathison, Richard S. Bradbury, Teerin Liewluck, Stefan Nicolau, John C. O’Horo, David Grunst, Marcus V. Pinto, Amy A. Swanson, Abinash Virk

**Affiliations:** Mayo Clinic, Rochester, Minnesota, USA (B.S. Pritt, T. Liewluck, J.C. O’Horo, D. Grunst, M.V. Pinto, A. Swanson, A. Virk);; ARUP Laboratories, Salt Lake City, Utah, USA (B.A. Mathison);; Federation University, Melbourne, Victoria, Australia (R.S. Bradbury);; Nationwide Children's Hospital, Columbus, Ohio, USA (S. Nicolau)

**Keywords:** Haycocknema perplexum, haycocknematosis, nematodes, parasites, imported infection, myositis, polymyositis, nematode infections, helminths, soft tissue infections, zoonoses, United States

## Abstract

We report an imported case of myositis caused by a rare parasite, *Haycocknema perplexum*, in Australia in a 37-year-old man who had progressive facial, axial, and limb weakness, dysphagia, dysphonia, increased levels of creatine kinase and hepatic aminotransferases, and peripheral eosinophilia for 8 years. He was given extended, high-dose albendazole.

*Haycocknema perplexum* is an enigmatic nematode that is a rare cause of human parasitic myositis (*1**‒**4*). Twelve cases have been reported since its initial description in 1998, all in humans (*1**‒**10*). The mode of transmission is unclear, but 9 patients reported contact with native wildlife in Tasmania or tropical regions of Queensland, Australia. We report an imported case of myositis caused by infection with this rare parasite.

## The Study

A 37-year-old man from New Zealand who had previous long-term residence in Australia came to the Mayo Clinic (Rochester, MN, USA) because of an 8-year history of progressive weakness, muscle atrophy, and 32-kg weight loss. Onset was gradual, first involving the pectoralis and biceps brachii, then neck, facial, and distal limb muscles. Additional symptoms included dysphagia, dysphonia, and dyspnea on exertion. Laboratory testing showed peripheral eosinophilia (5%, reference value <3%), and an increased level of creatine kinase (maximum ≈2,000 U/L, reference range 39–308 U/L). Toxoplasmosis had originally been suspected based on finding a possible *Toxoplasma gondii* cyst on muscle biopsy 1 year after symptom onset, but his weakness progressed despite trimethoprim/sulfamethoxazole therapy, and *T. gondii* serologic test results were negative. Prednisone therapy worsened his symptoms.

**Figure 1:**
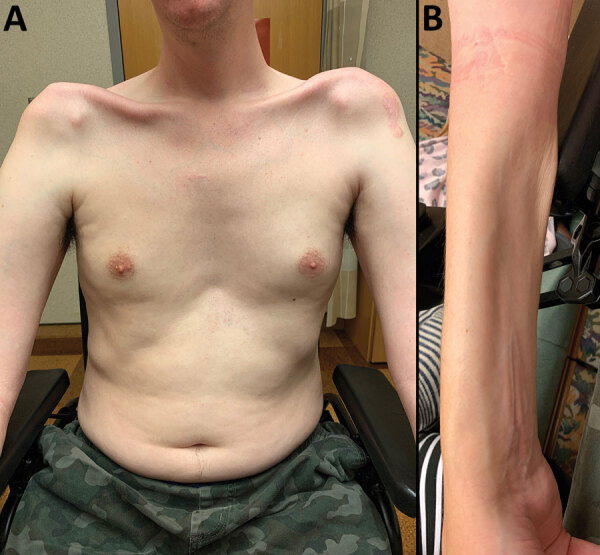
Physical manifestations of a patient who had imported *Haycocknema perplexum* infection, United States. Images show profound atrophy of the pectoralis and deltoid (A) and the forearm flexor musculature (B).

He became wheelchair-bound 7 years after onset of symptoms. He had lived in coastal northern Queensland (Mackay region), Australia from ages 8–20 years, where he had extensive bush exposure but denied bush meat consumption. Neurologic findings included profound asymmetric weakness predominantly affecting proximal upper and lower limbs, neck flexors, and sternocleidomastoids (Figure 1). He also had asymmetric scapular winging, severe weakness of the frontalis, and mild weakness of orbicularis oris.

**Figure 2:**
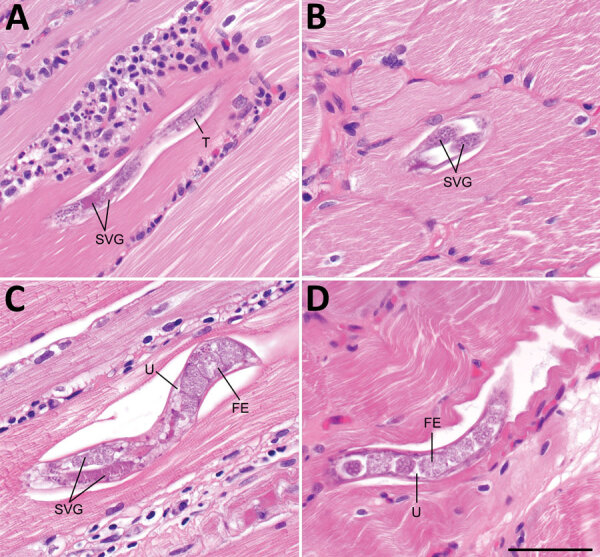
Histologic section of muscle tissue from the left deltoid of a patient who had imported *Haycocknema perplexum* infection, United States. A) Male *H. perplexum* in longitudinal section; B) anterior region of female *H. perplexum* in transverse section; C) anterior and midbody regions of gravid female; D) posterior region of gravid female. Scattered necrotic and regenerating fibers and dense inflammatory exudates were also observed. FE, fertilized eggs; SVG, subventral glands; T, testis; U, uterus. Scale bar indicates 50 μm.

A formalin-fixed, paraffin-embedded muscle tissue from a previous muscle biopsy specimen was obtained, and additional sections showed nonencapsulated male and gravid female nematodes within muscle fibers consistent with *H. perplexum* (Figure 2). The presence of adult worms enabled trichinellosis to be definitively excluded because only the larval stage of *Trichinella* sp. is detected in muscle. Attempts at molecular amplification of the cytochrome c oxidase subunit 1 and 18S rRNA genes as described (*2*,*8*) from archival formalin-fixed, paraffin-embedded tissue were unsuccessful.

The patient was prescribed a 3-month course of albendazole (400 mg 2×/d). Nineteen months after completing albendazole, the patient reported no further deterioration. However, his muscle power did not improve. Creatinine kinase levels decreased to within the reference range.

## Conclusions

*Haycocknema perplexum* is an enigmatic and presumably zoonotic nematode. Clinical histories of affected patients indicate that contact with wilderness or marsupial wildlife in Australia (n = 6) and consumption of bush meat (n = 4) might be associated with infection, but this possibility has not been confirmed (Table). The phylogenetic position of *H. perplexum* is unresolved, but it appears to be intermediary between Oxyuridomorpha (e.g., *Enterobius vermicularis*) and Ascaridomorpha (e.g., *Ascaris lumbricoides*) (*2*). All cases of haycocknematosis to date have originated in Australia, specifically in the tropical north of Queensland and Tasmania (Table). Nonhuman animal hosts are unknown. The route of human infection is also unknown but is presumed to be linked to consumption of, or contact with, mammalian wildlife. Because females are ovoviviparous (eggs hatch in utero within the female worm), infection caused by the ingestion of embryonated eggs is unlikely. With an apparent single-host (monoxenous) life cycle, an arthropod vector is also unlikely. Ongoing release and maturation of larvae results in persistent infections.

**Table:** Characteristics of 13 case-patients who had *Haycocknema perplexum* (haycocknematosis) infections*

Case-patient (reference)	Year of diagnosis	Country of diagnosis	Age, y/sex at diagnosis	Travel	Flora/fauna exposures	Duration of symptoms, y	Weakness	Weight loss, kg	Rash	Eosinophils, × 10^9^/L	CK, U/L	AST, U/L	ALT, U/L	ESR, mm/h	Treatment	Outcome
1 (*1**,**5**,**6*)	1994/1998†	NZ	33†/F	TAS,† NZ, Asia, Europe, Africa	Native flora (botanist), bush meat consumption	5	Dp, PLE, PUE	NA	Y	0.17–0.8	5,532	82–228	92–5532	NA	ALB. 400 mg, 2×/d for 8 weeks	Near full recovery
2 (*1*)	1996	AUS	48/M	TAS; trip Far North QLD and NT 5 earlier	NA	1.5	PLE, PUE	7	Y	2.0	1,586	84	197	26	5 weeks: mebendazole, 600–900 mg 3×/d; ALB 400 daily	Partial recovery
3 (*7*)	2004	AUS	61/M	TAS, Mackay (QLD)	NA	3	Da, Dp, F, Gen	NA	N	High, NOS	1,263	NA	NA	NA	ALB, 9 weeks	Died from complications
4 (*7*)	2005	AUS	23/F	Far North QLD, travel in WA, NSW and Victoria over previous 3 y	NA	2	DLE, Dp, PLE	18	N	1.1	1,370	52	60	50	ALB 400 mg 2×/d for 8 weeks	Partial recovery
5 (*7*)	2006	AUS	61/M	Mackay (QLD)	Exposure to native wildlife NA; No bush meat consumption	2	Dp, Gen	NA	N	1.36	1,230	67	69	NA	ALB, for 8 weeks	Partial recovery
6 (*9*)	2011	AUS	50/M	TAS, Ireland	Native wildlife; consumption of bush meat and unfiltered water	2	Dp, F, NE, NF, PLE>DLE, PUE>DUE	10	N	WNL	6,218	NA	92–152	NA	ALB, 400 mg 2×/d for 12 weeks	Near full recovery
7 (*3*)	2012	AUS	80/F	AUS, TAS, Asia, South America, Africa	Native wildlife (animal carer); bush meat consumption NA	1.5	PLE > DLE, PUE > DUE	5	N	0.7	270	NA	NA	15	ALB, 400 mg 2×/d for 12 weeks	No functional improvement
8 (*2*)	2014	AUS	30/M	North QLD, Mackay, Darling Downs (QLD)	No native wildlife, no bush meat consumption	2	Da, Dp, PLE > DLE, PUE > DUE	20	N	1.24	3,400	81–118	142–291	NA	ALB, 400 mg 2×/d for 16 weeks	Near full recovery
9 (*2*)	2014	AUS	72/M	Far North QLD, WA, TAS	Native wildlife (hunter), bush meat consumption	>2	Gen	NA	N	2.44	2,082	NA	NA	NA	ALB, 400 mg 2×/d for several weeks	Partial recovery
10 (*8*)	2016	AUS	37/M	TAS, Victoria	Native wildlife (hunter); bush meat consumption	2	PLE(A)	NA	N	0.54	3,636	94	139	NA	ALB, 400 mg 2×/d for 12 weeks	Partial recovery
11 (this study)	2019	USA	37/M	Mackay (QLD)	Native wildlife; no bush meat consumption	>8	Dp, Fa, NF, PLE (A) > DLE, PUE > DUE	32	N	0.16 (5%)	2,000	25‡	33‡	NA	ALB, 400 mg 2×/d for 12 weeks	Stable, no further deterioration at 19 mo after treatment
12 (*11*)	2021	AUS	≈40/M	North QLD, TAS	Limited wildlife, bush meat consumption	4	N	NA	N	2.1	2,530	65	94	NA	ALB, 400 mg 2×/d for 12 weeks	Remained asymptomatic
13 (*11*)	2021	AUS	≈20/F	North QLD, Brisbane, Thailand	No known exposures	3	DLE, NF, PUE	5	N	1.9	3,162	50–80	45–95	NA	ALB, 400 mg 2×/d for 12 weeks	NA

*A, asymmetry; ALB, albendazole; ALT, alanine aminotransferase; AST, aspartate aminotransferase; AUS, Australia; CK, creatinine kinase; Da, dysarthria or dysphonia; DLE, distal lower extremity; Dp, dysphagia; DUE, distal upper extremity; ESR, erythrocyte sedimentation rate; Fa, facial; Gen, generalized weakness not otherwise specified; N, no; NA, not available; NE, neck extensors; NF, neck flexors; NOS, not otherwise specified; NSW, New South Wales; NT, Northern Territory; NZ, New Zealand; PLE, proximal lower extremity; PUE, proximal upper extremity; QLD, Queensland; TAS, Tasmania, USA, United States; WA, Western Australia; WNL, within normal limits, Y, yes. †Patient 1 was initially misdiagnosed as having trichinosis in 1994 and was retrospectively diagnosed as having haycocknematosis in 1998. Despite an extensive previous travel history, her most extensive wildlife exposures were in Tasmania and the Australian Northern Territory. ‡AST and ALT levels for patient 11 were mildly increased in 2011, but exact values are not available. These levels were not increased during his most recent presentation.

Of the 13 known case-patients (Table), 12 had weakness and muscle wasting, 7 had dysphagia, and 2 had dysarthria or dysphonia. One case was discovered incidentally during evaluation of low back pain; the patient was otherwise asymptomatic. All case-patients had increased levels of creatinine kinase (270–6,218 U/L). Peripheral eosinophilia was observed in 12 (92%) of 13 patients. Myalgias, unintentional weight loss, increases in erythrocyte sedimentation rates, and mild-to-moderate increases in levels of liver aminotransferases were also common. Needle electromyography findings were available for 8 patients (patients 2, 4, 7, 8, 10, 11, 12, and 13). Except for patients 10 and 12, who had ambiguous or limited findings, the remaining patients had myopathic motor unit potentials. Results of nerve conduction studies were within reference ranges when described. The time from symptom onset to diagnosis ranged from 1.5 to 8 years, with the case-patient in this study having the longest known timeframe.

Seven patients had received corticosteroids at some point in their illness for a presumptive diagnosis of polymyositis, during which time most experienced progressive deterioration, including our patient. All patients were given extended, high-dose albendazole therapy, and 7 patients had a partial to near complete recovery. One patient (*7*) died from complications resulting from corticosteroid administration, mechanical ventilation, and a prolonged stay in the intensive care unit.

Diagnosis of haycocknematosis is based primarily on histopathologic features. The morphologic characteristics of *H. perplexum* nematodes in histopathologic preparations include a thin cuticle, meromyarian/platymyarian musculature, amphidelphic uteri (females), lateral bacillary bands (especially conspicuous in immature females), and conspicuous subventral glands (*10*). There are no cephalic inflations or lateral alae. Adult males, adult females, and larvae might be observed in muscle specimens, but never in ex utero eggs. Adults often have an undulating, serpentine morphology, which is parallel with the muscle fibers. Male worms have a maximum width of 15 μm (range 14–15 μm), and female worms have a maximum width of 36 μm (range 15–36 μm) (*10*). Larvae are similar in size to adult males, have a maximum width of 15 μm (range 12–15 μm) (*10*), and complete their lifecycle in the host.

Although other parasites in biopsy specimens include *Trichinella* spp., *Strongyloides stercoralis*, and *Halicephalobus gingivalis*, these parasites can be differentiated by morphologic, clinical, and epidemiologic features. *H. perplexum* and other nematodes might also be potentially confused for tissue cysts of *Toxoplasma gondii* and *Sarcocystis* spp. when seen in cross-section (as in our case-patient), but this finding can usually be resolved by examining deeper sections from the tissue block to identify additional parasite forms.

A PCR was developed that enabled diagnosis of the 10th case of *H. perplexum* nematode infection from a muscle biopsy in the absence of visible nematodes (*2*,*8*). This PCR was unsuccessful when performed for our case-patient. However, this result is not unexpected, given the age of the block (7 years at time of testing) and the relatively large sizes of the PCR amplicons (400 bp for cytochrome c oxidase subunit 1 and 830 bp for 18S rRNA).

It is unknown why to date human cases appear to have been acquired only in Tasmania or the northern regions of Queensland. Molecular sequence data demonstrate that the strains from Queensland and Tasmania belong to the same species (*2*). Infections might occur in other areas of the East Coast of Australia, or wider mainland Australia, but these infections have not been detected because of lack of awareness and difficulty in diagnosis.

The optimal antimicrobial drug management for treatment of haycocknematosis is unknown. Our patient was given albendazole based on experiences from previously reported cases. In 1 case, viable nematodes were still observed after 4 weeks of treatment, but not after 9 weeks (*7*). Additional studies are needed to determine the most efficacious antiparasitic treatment for haycocknematosis.

Patients who have *H. perplexum* parasitic myositis might be a diagnostic challenge to clinicians and pathologists, particularly when seen outside disease-endemic regions. The disease is progressive, potentially life-threatening, and might persist for >8 years with a delayed diagnosis, as shown by our case-patient. A high degree of suspicion is required to diagnose this treatable mimic of muscular dystrophy and inflammatory myopathy, and to avoid harm through corticosteroid treatment. Additional studies are needed to clarify the exposure risks, parasite life cycle, disease prevention, and treatment.
